# Progress on the Implementation of Environmental Surveillance in the African Region, 2011-2016

**Published:** 2018-07-02

**Authors:** Nicksy Gumede, Joseph Okeibunor, Ousmane Diop, Maryceline Baba, Jacob Barnor, Salla Mbaye, Johnson Ticha, Goitom Weldegebriel, Humayun Asghar, Pascal Mkanda

**Affiliations:** 1 WHO Regional Office for Africa, Brazzaville, Republic of Congo; 2 WHO Head Quarters, Geneva, Switzerland; 3 Intercountry support teams (ISTs) in East and Southern Africa, Harare, Zimbabwe; 4 WHO Regional Office for East and Mediterrian, Cairo, Egypt

## Abstract

**Objective:**

This article summarises the progress made since the introduction of environmental surveillance in the African Region.

**Method:**

Country selection was based on the poor AFP performance indicators i.e. Non polio AFP rate and stool adequacy. It was recommended that any country not meeting the required indicators should consider environmental surveillance activity as an additional tool to support AFP surveillance. The sites selection considered proximity to the target population, the size of the population to be sampled and the sensitivity of the sampling site.

**Results:**

One hundred and fifty three sites have been established in Africa since 2011. In 2011, Nigeria was first country to introduce environmental surveillance and currently with of 59 validated sites, followed by Kenya in 2013 validating and sampling 9 sites and Angola 4 active sites in 2014. In 2014, Cameroon introduced ES and 31 sites followed by Niger with 9 sites and Madagascar with 23 sites. Later in the same year, Chad introduced ES activity and 4 active sites were selected. In 2015 Senegal introduced 3 sites, Guinea and Burkina Faso introduced 4 sites each., and. In 2016, a total of 179 Sabins, 36 Sabin 2s, 196 non polio enteroviruses (NPEV) and 1 vaccine-derived polioviruses (VDPV) were reported in Nigeria. Cameroon and Chad isolated 14 and 4 Sabins and 72 and 40 NPEV respectively. In Madagascar a total of 39 Sabins, 11 Sabin 2s and 277 NPEV were isolated. In other countries a majority of NPEV were isolated (data not shown).

**Conclusion:**

This report describes the progress and expansion of environmental surveillance that contributed to the identification of polioviruses from the environment and the interruption of wild poliovirus transmission in the African Region.

## Introduction

Poliovirus, a member of the Enterovirus genus of the family Picornaviridae, is the cause of paralytic poliomyelitis which occurs only in a small proportion (<1%) of poliovirus infections of susceptible individuals^1^. Poliovirus has a predilection for the motor nerve cells of the brain and spinal cord, and infection results in their destruction and paralysis of the muscles supplied by the affected cells^2^. Some infected people experience minor illness of several days and a symptom-free interval of 1 to 3 days, followed by acute onset of flaccid paralysis with fever. Within few days, paralysis which is usually asymmetric, progresses depending upon the sites of virus replication in the central nervous system and may affect skeletal muscles (spinal poliomyelitis), respiratory muscles (bulbar poliomyelitis), or both (bulbo-spinal poliomyelitis)^3^.

Since the launch of the Global Polio Eradication Initiative (GPEI) in 1988, cases of poliomyelitis have dropped by more than 99%^4^–^6^. No type 2 wild poliovirus (WPV) has been identified since 1999^7^ and the remaining serotype 1 is limited to endemic countries and in outbreak settings.

The World Health Organization strategy for monitoring the wild polioviruses and mutated vaccine polioviruses is to identify virus isolates from stool samples taken from Acute Flaccid Paralysis (AFP) patients (sampling contacts is also supplemental to AFP surveillance). Environmental surveillance serves as an additional method to monitor the transmission of poliovirus by testing sewage samples, which may contain polioviruses in human faeces^8^.

The some of the reasons for conducting environmental surveillance are the detection of the circulating polioviruses (PV) as a supplementary method for acute flaccid paralysis surveillance for suspected polio cases. It can a) assist in identifying residual wild poliovirus (WPV) transmission in endemic areas, b) provide an early indication of new poliovirus importations into polio-free areas and c) confirm the presence of vaccine-related virus following a vaccination campaign using oral polio vaccine (OPV). Environmental surveillance playa a critical role in detecting the ongoing circulation of wild polioviruses and circulating vaccine derived polioviruses, monitoring the disappearance of Sabin-related viruses, monitoring around poliovirus containment facilities and providing evidence for global certification^9^,^10^.

This report looks at the progress and the expansion of environmental surveillance in the African since its initiation in 2011.

## Methods

### Selection criteria

The following factors are used to determine the prioritization and rationale for selecting countries and sites. The sites expansion or discontinuation is also considered when monitoring environmental surveillance for polioviruses. The selection of sites should be informed by whether they are endemic locations; polio-free districts adjacent to endemic districts; in the same or adjacent countries; or areas with recent or recurrent importation, re-establishment of transmission or history of silent transmission in the face of inadequate AFP surveillance indicators.

The country must have a capacity for initial processing of the samples and for transporting them rapidly to the laboratory. The laboratory capacity must first be sufficiently increased to be able to cope with the high workload. When sites are selected, consideration should be made for households that are equipped with water closets connected to a converging sewer network allowing collection of downstream samples that represent a large number of people living in the catchment area. Also, when selecting sites, consideration should be made to select areas without chemicals from industrial wastes that may be detrimental to poliovirus stability or be toxic to cell cultures and interfere with poliovirus replication. Lastly selected sites should represent selected high-risk populations.

Using these criteria, the following Regional polio laboratories under Polio Laboratory Network (Polio LabNet) have been selected to carry out testing of environmental samples: Nigeria, Cameroon, Kenya, Madagascar, Senegal, and South Africa. Due to increase of demand of the establishment of environmental surveillance, more laboratories have been considered to perform ES activity such as laboratories in the following countries: Ethiopia, Algeria, Central Africa Republic, Cote d’Ivoire, Uganda and Zambia. Nigeria will continue serving its population, Senegal will focus on West African countries, Cameroon on Central African countries, Kenya the Horn of Africa and South Africa the southern African countries and countries they serve as their national laboratory for AFP. South Africa will be carrying the sequencing task for the entire Region while Madagascar will support its population.

Proposed additional countries to expand or to initiate environmental surveillance in 2017 includes Uganda, South Sudan, Burundi, Ethiopia, Algeria, Guinea Bissau, Guinea, Equatorial Guinea, Gabon, DR Congo, Sierra Leone, Liberia, Cote d’Ivoire, Mali, Tanzania, Zambia, Mozambique, South Africa and the Central African Republic.

### Sample collection and processing

The grab method is recommended by WHO whereby an amount of raw sewage is collected at a selected sampling site with the time of collection noted (preferably early in the morning to avoid heat that might decrease the viability of the virus). The larger the volume of sewage analysed, the higher the theoretical sensitivity to detect poliovirus circulation in the source population. Processing of sewage specimens is performed by using the two-phase separation method. A small volume lower phase is created by adjusting the concentrations of the stock solutions and relative volumes of the two polymer solutions. Concentrated elements are attracted to the interphase and lower phases, including polioviruses and several other enteroviruses.

## Results

Environmental surveillance started in the African Region in July 2011 in Nigeria. Kano state was the first to introduce environmental surveillance and it later expanded to Sokoto, Lagos, Kaduna, the Federal Capital Territory (FCT), Kebbi, Katsina, Jigawa, Borno, Yobe, Adamawa, Rivers, and Osun. The additional states were Gombe, Bauchi and Gombe. In total, 59 sites of environmental surveillance have been established in Nigeria since its inception in 2011. In 2013, Kenya started its first environmental sampling as a pilot study to supplement AFP surveillance after a Horn of Africa WPV1 outbreak in May 2013. Two sites were identified and sampled in 2013, Kamukunji and Kibera. Furthermore, an expansion in areas of the northeastern counties and other risk areas for WPV transmission such as Mathare, Patel flats, Kisumu Polytechnic, Mlango was Papa, Kipevu, and Bulla (unpublished data). In total nine sites in two counties have been identified in Kenya.

**Figure 1:**
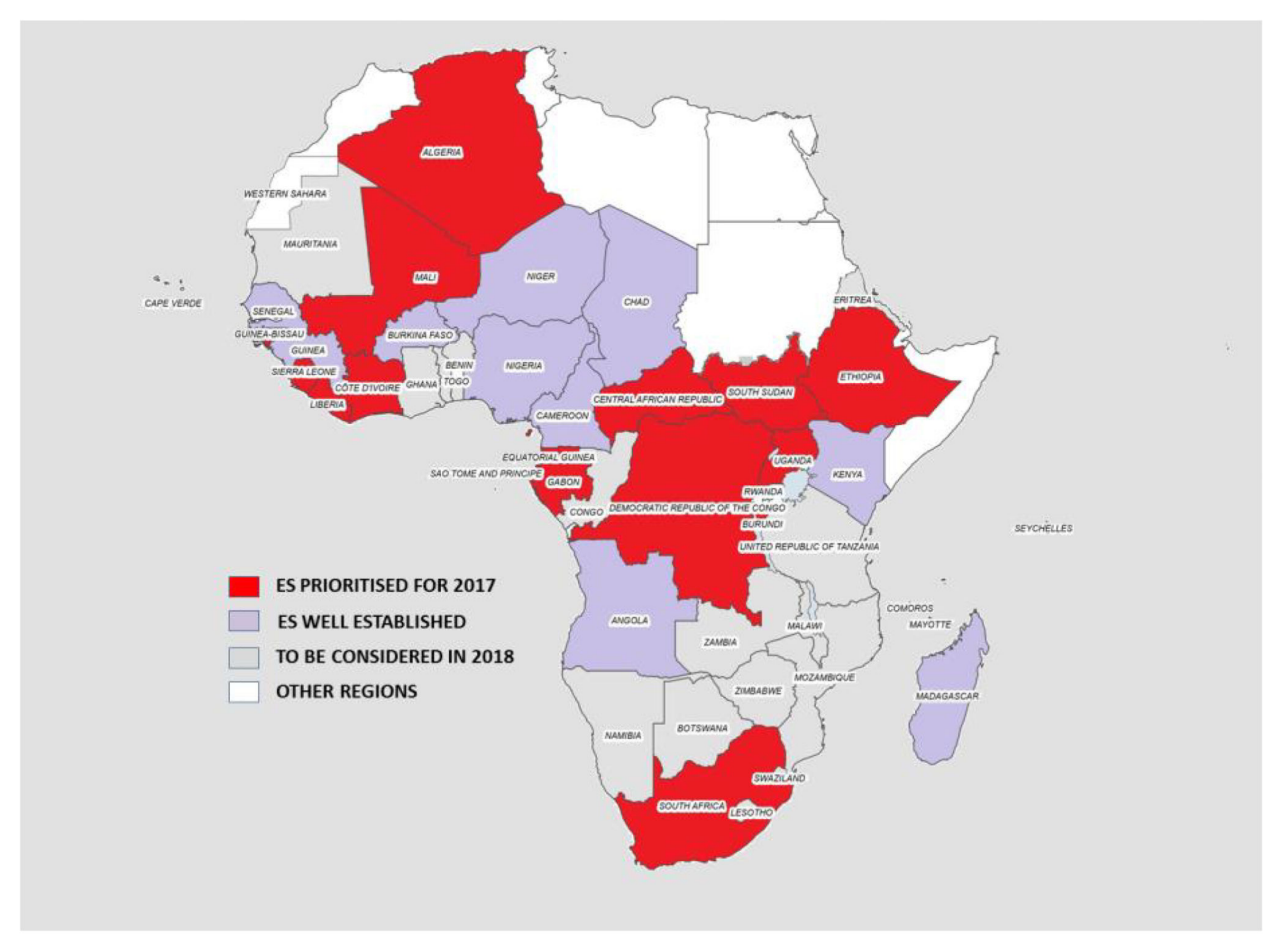
Map of environmental surveillance expansion Plan in the African Region

As from 2014, additional countries were added to the African Region expansion plan such as Angola with 4 sites, Madagascar with 23 sites, Cameroon with 31 sites, Chad with 4 sites, Senegal with 3 sites, Niger 9 sites, Burkina Faso with 4 sites. Four sites were recently selected, validated and sampled in Guinea. Two countries such as the Democratic Republic of the Congo and Mali have identified potential sites for sampling and the training for processing of the sewage samples is due first quarter of 2017. The Regional expansion plan has added more countries based on the poor performing on the AFP indicators that are not meant such as non-polio AFP rate and also stool adequacy (Figure 1).

**Figure 2:**
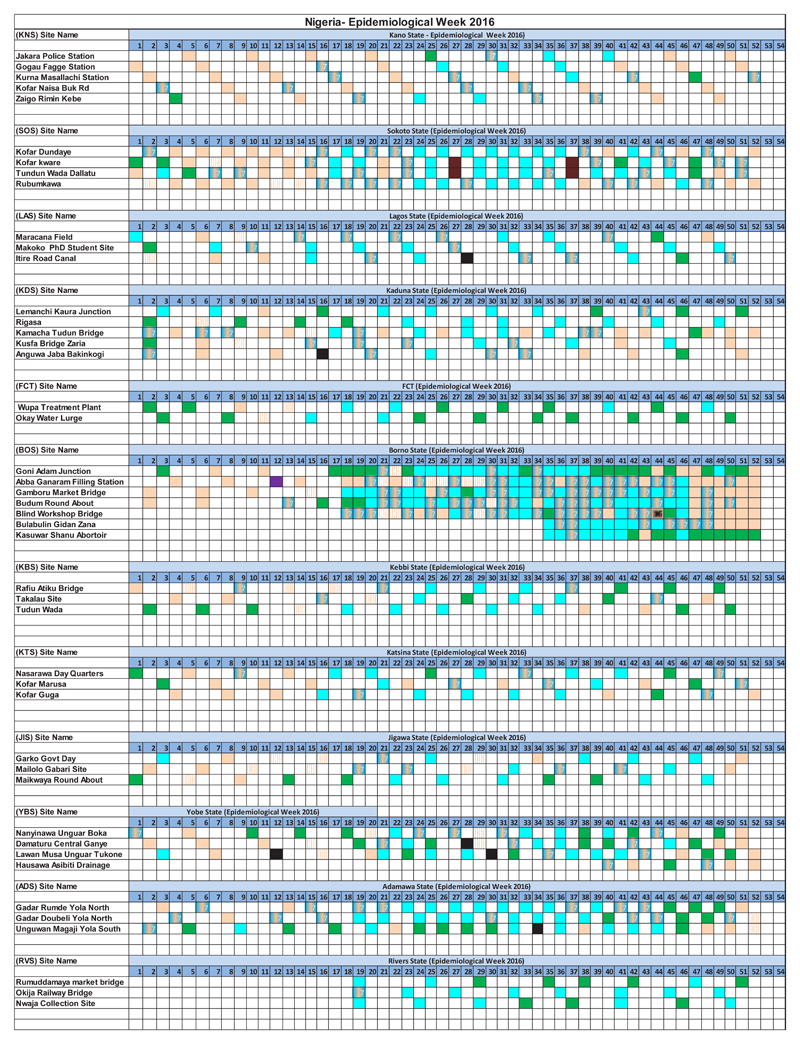

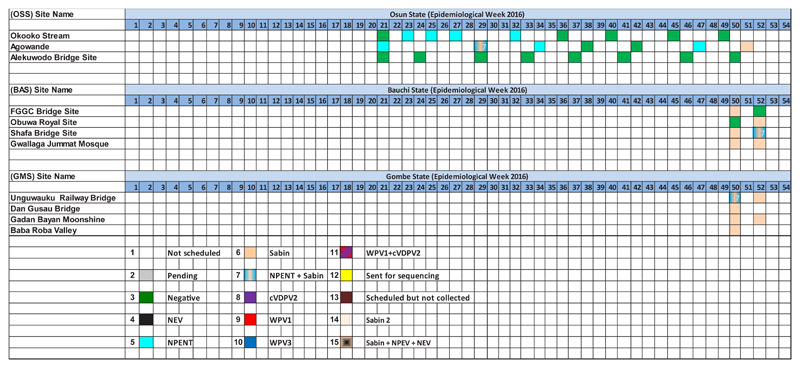
Environmental results for Nigeria

**Figure 3:**
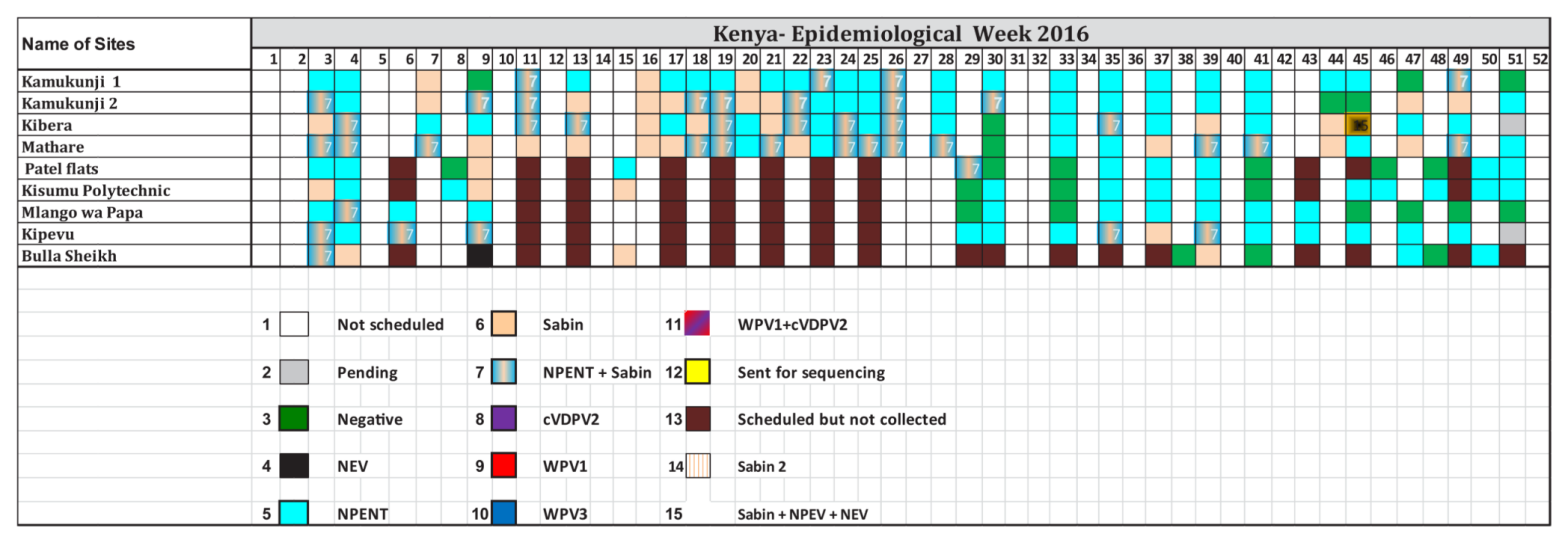
Environmental results for Kenya

From 2011 to 2016, environmental surveillance detected 126 cVDPV2, 10 WPV1, and 3 WPV3 in the African Region. In 2013, 3 WPV1 were detected in Kano and Sokoto States, and a total of 33 cVDPV2 were identified in states of Nigeria (2 in Kaduna, 8 in Kano, 1 in Katsina, 10 in Sokoto and 12 in Borno). One WPV1 was reported in Kaduna State. In 2016, a circulating vaccine poliovirus type 2 (cVDPV2) was identified in Abba Ganaram Filling Station site of Borno, 179 Sabin viruses, 36 Sabin viruses and 196 non polio enteroviruses were reported (Figure 2). As this incident happened during the trivalent oral poliovirus vaccine (tOPV) to bivalent oral poliovirus vaccine (bOPV) switch period, there was a need to official request monovalent OPV2 (mOPV2) to respond to this outbreak hence and identification of Sabin 2 viruses after switch period. Other countries such as Cameroon and Chad isolated 14 and 4 Sabins and 72 and 40 NPEV respectively. In Madagascar a large number of NPEV were identified totaling 277 NPEV followed by 39 Sabins, 11 Sabin 2 viruses. In October 2013, a wild poliovirus was identified from a sewage sample collected in Kamukunji sub-county pointing to silent transmission or surveillance gaps. During the first quarter of 2016, a cVDPV2 was from sewage samples collected in December 2015. Outbreak response was conducted with success. At the time, the laboratory experienced a high increase in workload as a results some samples were diverted to laboratories at the US Centers for Disease Control and Prevention (CDC), in Atlanta USA. The most sites selected were not sampled due to technical problems in the field (Figure 3).

**Figure 4:**
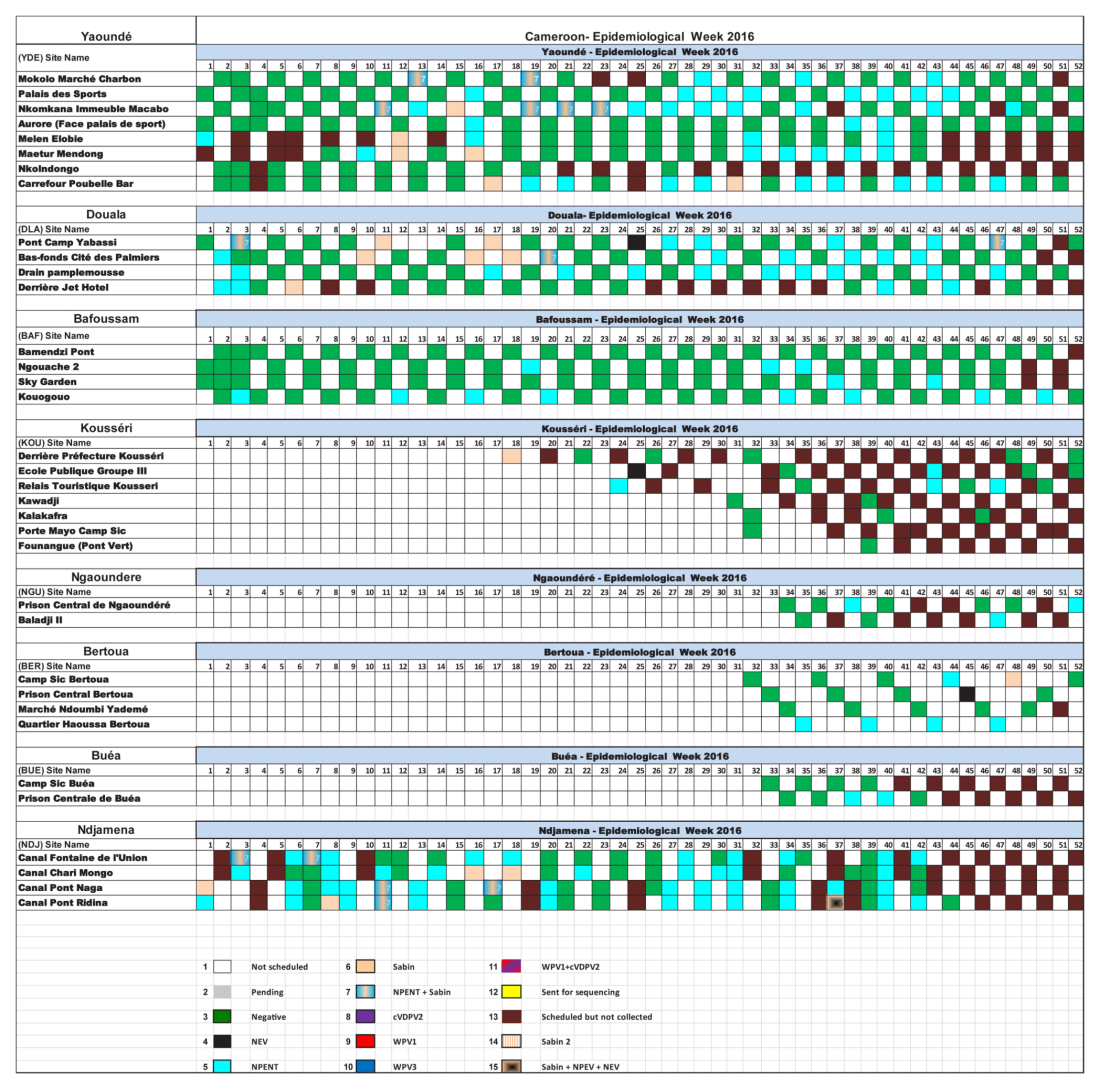
Environmental results for Cameroon

Cameroon has been supporting both Cameroon and Chad in processing sewage for the environmental surveillance. In total, twenty sites and four sites have been established in Cameroon and Chad respectively. The results have shown that some sites have not revealed any type of poliovirus, therefore, there is a need of closing down such sites (Figure 4). All four sites in Angola revealed no wild poliovirus, but 1 non polioenterovirus (NPEV) has been identified in Rio Cambamba Bairro (Kilamba Kiaxi) site.

## Discussion

As a supplementary method to AFP surveillance for the Global Polio Eradication Initiative, environmental surveillance is of great importance in investigating the circulation of wild polioviruses and vaccine derived polioviruses (11 and has led to the identification of wild polioviruses, Sabin viruses, non-polio enteroviruses and circulating derived polioviruses.

The African Region remains an area of attention on its large susceptible population, with a higher chance of contracting poliomyelitis and a greater likelihood of the spread of polioviruses compared with other regions. Vulnerable groups within a population may contribute to poliovirus circulation because of prolonged shedding. The oral poliovirus vaccines (OPV) and the inactivated poliovirus vaccine (IPV) have made poliomyelitis a preventable and eradicable disease, however, they cannot protect against poliovirus infection or shedding^12^. The screening of sewage material has been recognized as a useful tool for monitoring the circulation of the wild poliovirus and the vaccine-derived polioviruses.

Due to the usage of mOPV2 as part of outbreak response in Nigeria, the following states; Sokoto, Kaduna, Borno, Kebbi, Jigawa and Yobe have confirmed the isolation of the Sabin 2 virus which is an indication of the sensitivity of the sites. The last Sabin 2 was reported on the second week of August 2016 in Borno state.

It was noted that Cameroon had a serious problem with the field sampling on the set date due to the absence of funds to pay sample collectors from the field staff. Measures had been put in place as a result the routine sampling has been ongoing without any delays in 2017.

Besides the use of environmental surveillance in the global eradication programme for polio, it can be used in detecting the circulation of other human viruses such as adenoviruses, caliciviruses, rotaviruses, and noroviruses^13^-16. Environmental surveillance can help assess the efficacy of policy decisions in preventing the spread and transmission of poliovirus and poliomyelitis diseases. The results presented in this report confirm the importance of environmental surveillance in the Global Polio Eradication Initiative Programme.

Experiences from Nigeria confirm that environmental surveillance can detect the introduction and silent circulation of WPV and VDPV. Likewise, ES with its greater sensitivity than AFP surveillance can monitor the presence of poliovirus circulation. Furthermore, ES can be used to monitor the efficacy of immunization interventions and further confirm any usage of OPV2 should Sabin 2 or VDPV2 be identified in the environmental surveillance samples.

Due to the regional expansion plan and poor AFP surveillance there is a need of increasing more countries to initiate environmental surveillance and be supported by newly developed environmental surveillance Polio laboratories.
